# Ketone Body Improves Neurological Outcomes after Cardiac Arrest by Inhibiting Mitochondrial Fission in Rats

**DOI:** 10.1155/2022/7736416

**Published:** 2022-07-07

**Authors:** Yunke Tan, Jie Zhang, Qiulin Ge, Xiangshao Fang, Fengqing Song, Tao Yu, Longyuan Jiang, Yongli Wei, Peng Wang

**Affiliations:** ^1^Department of Emergency Medicine, Sun Yat-sen Memorial Hospital, Sun Yat-sen University, Guangzhou 510120, China; ^2^Institute of Cardiopulmonary Cerebral Resuscitation, Sun Yat-sen University, Guangzhou 510120, China; ^3^Grade Three Laboratory of Traditional Chinese Medicine Preparation of the National Administration of Traditional Chinese Medicine, Affiliated Hospital of Shandong University of Traditional Chinese Medicine, Jinan 250014, China

## Abstract

Ketone bodies including *β*-hydroxybutyrate (*β*-HB) have been proved the therapeutic potential in diverse neurological disorders. However, the role of *β*-HB in the regulation of neurological injury after cardiac arrest (CA) remains unclear. We investigated the effect of *β*-HB on brain mitochondrial dysfunction and neurological function after CA. A rat model of CA was established by asphyxia. The rats were randomly divided into three groups: sham group, control group, and *β*-HB group. Animals received 200 mg/kg *β*-HB or same volume vehicle at 10 minutes after return of spontaneous circulation by intraperitoneal injection. Neurological function was evaluated by neurologic deficit score and Y-maze. Neuronal cell loss and apoptosis were detected through hematoxylin-eosin staining, Nissl staining, and TdT-mediated dUTP nick-end labeling assay. Oxidative stress levels were determined by immunohistochemical staining of 4-hydoxynonenal and 8-hydroxy-2′-deoxyguanosine. Furthermore, mitochondrial ultrastructure of brain cells was observed by transmission electron microscopy. In addition, the protein expression levels of Bak, caspase 3, gasdermin D, caspase 1, brain-derived neurotrophic factor, dynamin-related protein 1 (Drp1), and phospho-Drp1 (ser616) were measured. We found that neurological function and survival rate were significantly higher in the *β*-HB group compared with the control group. *β*-HB also reduced neurons death and neurological oxidative stress after CA. Moreover, *β*-HB reduced neurological injury from apoptosis and pyroptosis after CA. In addition, *β*-HB maintained the structural integrity of brain mitochondria, prevented mitochondrial fission, and increased brain energy metabolism after CA. In conclusion, *β*-HB beneficially affected the neurological function of rats after global cerebral ischemia, associated with decreased mitochondrial fission, and improved mitochondrial function. Our results suggest that *β*-HB might benefit patients suffering from neurological dysfunction after CA.

## 1. Introduction

Although a great effort has been done to improve resuscitation techniques, morbidity and mortality caused by cardiac arrest (CA) remain high all over the world [1, 2]. The most common causes of CA are asphyxia and ventricular fibrillation. Cardiopulmonary resuscitation (CPR) is the most essential treatment of CA. However, even timely CPR is carried out, most patients still fail to achieve the return of spontaneous circulation (ROSC). Moreover, those patients that achieve ROSC suffer from post-CA syndrome, including neurological injury and myocardial dysfunction. Brain injury is primarily responsible for the deaths (almost two-thirds) in the post-CA period [3]. However, except for the targeted temperature management, there is no other treatment that has been proved to be beneficial to brain injury after CA in clinic, especially beneficial drugs.

Mitochondrial dysfunction plays a key role in brain ischemia/reperfusion (I/R) injury pathogenesis after CA [4]. Mitochondria change their number and shape by fusion and fission continuously, and recent studies have demonstrated that the balance of fusion and fission is important for mitochondrial function [5]. Increased mitochondrial fission has been considered harmful and is one reason for damaged energy metabolism, increased reactive oxygen species (ROS), and released detrimental cytokines [6]. Less of oxygen and excessive mitochondrial fission during ischemia and refusion lead to a dramatic decrease in generation of adenosine triphosphate (ATP) and ROS accumulation. ATP depletion and ROS amassing contribute to brain I/R injury. Therefore, inhibiting mitochondrial fission and enhancing ATP supply might be attractive methods to reduce brain injury after CA.

Ketone bodies (KBs) are small molecules that consists of acetoacetate, *β*-hydroxybutyrate (*β*-HB), and acetone, and most of them are produced from lipid *β*-oxidation in liver cells. *β*-HB is the most abundant KB, which constitutes about 70% of the circulating KB pool. The plasma concentration of *β*-HB in humans ranges from 0.05 to 0.1 mM under physiological conditions, whereas the concentration levels can reach 5-7 mM in conditions of starvation, ketogenic diet, prolonged exercise, or insulin deficiency. In diabetic patients, the plasma concentration can even exceed 20 mM, which is an indicative concentration of diabetic ketoacidosis [7]. Early studies showed that KBs act as signaling molecules which enhance mitochondrial function and endogenous antioxidant defense [8, 9]. Thus, although ketoacidosis is a pathological state, mild ketonemia proved beneficial in animal models, because of improving metabolic profile, prolonging lifetime, and improving neurological responses [10].

However, the effect of *β*-HB in the regulation of neurological injury after global cerebral ischemia has not been studied. In this study, we hypothesized that *β*-HB could alleviate brain injury after global cerebral ischemia by improving mitochondrial function. We therefore investigated the effects of *β*-HB on brain injury in a rat model of asphyxia-induced CA.

## 2. Materials and Methods

### 2.1. Animals and Drugs

All experiments were performed in male Sprague-Dawley rats weighting 387 to 463 g (Experimental Animal Center of Sun Yat-sen University, Guangzhou, China). All animals were housed in a specific pathogen-free room at 20°C to 22°C with 12 hours light/12 hours dark cycles and maintained on laboratory chow. The rats were cared for based on the guidelines of the National Institutes of Health Guide to the Care and Use of Laboratory Animals. All experimental protocols were approved by the Institutional Animal Care and Use Committee of the Sun Yat-sen University (SYSU-IACUS-2021-B1416). *β*-HB was purchased from Sigma-Aldrich (St. Louis, MO, USA) and was dissolved in phosphate-buffered saline (200 mg/mL).

### 2.2. CA Model and Experimental Procedures

The animals were fasted for 8 to 10 hours before the experiments. The rats were intraperitoneally injected pentobarbital sodium (45 mg/kg) for anesthesia. A 14-gauge cannula (Abbocath-T, USA) was intubated in the trachea. Advanced 23-gauge polyethylene catheter (Abbocath-T, USA) through the left femoral artery to monitor mean arterial pressure (MAP). The computer-based collection system (WinDaq acquisition system, USA) was used to measure and record the hemodynamic data continuously.

After instrumentation, asphyxia was induced by clamping the endotracheal tube. CA was defined as a MAP of ≤30 mmHg. After 8 minutes of untreated CA, CPR was started. Epinephrine (0.01 mg/kg) was injected into the femoral artery after 2-minute of precordial compression and repeated every 2 minutes. A 2 J biphasic waveform shock was performed when ventricular fibrillation appeared. ROSC was defined as the presence of a regular supraventricular rhythm with a MAP ≥ 60 mmHg and lasted at least 5 minutes. After a continuous 6 minutes of CPR, precordial compression was stopped if there was no return of ROSC.

The rats were divided into three groups randomly: sham group, control group, and *β*-HB group. All animals underwent the same surgery, but those in the sham group did not reduce CA. The rats in *β*-HB group were given 200 mg/kg of *β*-hydroxybutyrate by intraperitoneal injection at 10 minutes after ROSC, while the animals in control group were received the same volume of saline.

### 2.3. Neurologic Deficit Score

Neurologic deficit score (NDS) was determined on a 0-80 scale. 0 = death or brain death. 80 = no observed neurologic deficit. The NDSs were scored by two researchers who were unknown of the group identities.

### 2.4. Y-Maze Spontaneous Alteration

The Y-maze spontaneous alteration tests were performed using a Y-shaped maze of three equal length arms. Labeled the three arms of the maze A, B, and C. Make sure that the maze was clean and dry before the test. Place a camera above the Y-maze to record the experiment. Put the test animal into to the terminal part of the arm labelled A, facing to the center, then hold the base of the tail lightly. Leave the testing room gently, and let the rats explore the maze freely for 10 minutes. Stop recording, and place the animal back to its cage. An entry was defined as all four limbs of the rat were within an arm. Record number of all entries and alternations. Calculate the percent alternation with the following formula: Percent Alternation = [Number of Alternations/(Total number of arm entries − 2)] × 100. A high percentage meant good working memory because it indicated that the rat had remembered which arm it had entered already [11].

### 2.5. Hematoxylin-Eosin (HE) Staining, Nissl Staining, and TdT-Mediated dUTP Nick-End Labeling (TUNEL) Assay

The brain tissues were harvested at 24 hours after ROSC and embedded in paraffin, and 5 *μ*m thick sections were cut for examination. HE staining, Nissl staining, and TUNEL assay (Beyotime, Nantong, China) were performed for detection of neuronal injury in situ according to the manufacturer instructions. Six fields of vision (400x magnification) were selected randomly from each sample. HE-stained neurons in the hippocampal CA1 area and the TUNEL-positive neuronal cells in the cortex area were counted. The average optical density (AOD) of neuronal cells in the hippocampal CA1 area of Nissl staining was calculated using Image J software (National Institute of Health, United States) [12].

### 2.6. Measurements of *β*-HB, Lactate, and ATP Levels and NAD^+^/NADH Ratios

Serum and brain *β*-HB levels were measured using a *β*-HB Colorimetric Assay Kit (BioVision, Milpitas, CA, USA) as the manufacturer's instructions. Serum lactate levels were measured using a biochemistry analyzer (YSI, Yellow Springs, OH, USA). The ATP levels of brain tissue were measured using an Enhanced ATP Assay Kit (Beyotime, Nantong, China) as the manufacturer's instructions. The NAD^+^/NADH ratios were measured using a NAD^+^/NADH Quantification Colorimetric Kit (BioVision, Milpitas, CA, USA) as the manufacturer's instructions.

### 2.7. Detection of Oxidative Stress

To detect the oxidative stress of the brain tissue at 24 hours after ROSC, immunohistochemical staining of 4-hydroxynonenal (4-HNE) and 8-hydroxy-2′-deoxyguanosine (8-OHdG) was conducted. 4-HNE was conducted to assess lipid peroxidation while 8-OHdG was conducted to evaluate the extent of nucleic acid oxidation. For immunohistochemical staining, the brain sections were incubated with a primary monoclonal antibody against 4-HNE and 8-OHdG (Abcam, Cambridge, United Kingdom) overnight at 4°C. After primary antibody incubation, sections were washed three times for 10 minutes with phosphate-buffered saline. After washing, they were incubated with the corresponding secondary antibody for 2 hours at room temperature. Six fields of vision (400x magnification) of these sections were selected randomly and photographed with an optical microscope. For analysis, the AOD of 4-HNE and 8-OHdG were calculated using Image J software (National Institute of Health, United States). The ROS levels in the brain at 6 hours after ROSC were measured using a ROS assay kit (Genmed Scientifics, Wilmington, USA) as the manufacturer's instructions.

### 2.8. Western Blot Analysis

For immunoblotting at 24 hours after ROSC, the following antibodies were used: anti-Bak (rabbit, Servicebio), anti-caspase 3 (rabbit, Servicebio), anti-gasdermin D (GSDMD) (mouse, Santa Cruz), anti-caspase 1 (rabbit, Servicebio), anti-brain-derived neurotrophic factor (BDNF) (rabbit, Servicebio), anti-Drp1 (rabbit, Signalway Antibody), and anti-phospho-Drp1 (ser616) (rabbit, Signalway Antibody). Immunoreactivity was semiquantitatively calculated by Image J software (National Institute of Health, United States).

### 2.9. Transmission Electron Microscopy and Analysis

Brain tissues at 6 hours after ROSC were harvested and fixed in 0.1 mol/L phosphate buffer (pH 7.4) with 2.5% glutaraldehyde overnight at 4°C. Then, the samples were sectioned into 100 nm thick slices and fixed in 1% osmium tetroxide for 1 hour. After that, the sections were dehydrated, embedded in epon, and conventionally processed. The samples were examined under a transmission electron microscope (Tecnai G2, FEI, Hillsboro, United States) through a magnification of ×13500. Areas of mitochondria were calculated by Image J software (National Institute of Health, United States).

### 2.10. Statistical Analysis

All results were presented as mean ± standard deviation (SD). SPSS 20 for Windows (SPSS, Chicago, IL, United States) was used for statistical analysis. For continuous variables with normal distribution, the differences in means among the three groups were analyzed using one-way analysis of variance (ANOVA). When ANOVA showed significant differences, LSD post hoc tests were used to test the pare-wise comparisons between means. Neurological deficit scores were analyzed using the Kruskal-Wallis test followed by Dunnett post hoc test. The survival rates were compared by Kaplan-Meier survival analysis. In all analysis, a 2-sided *p* < 0.05 was considered statistically significant.

## 3. Results

### 3.1. *β*-HB Improved Neurological Outcomes and Survival after Cardiac Arrest

**Figure 1:**
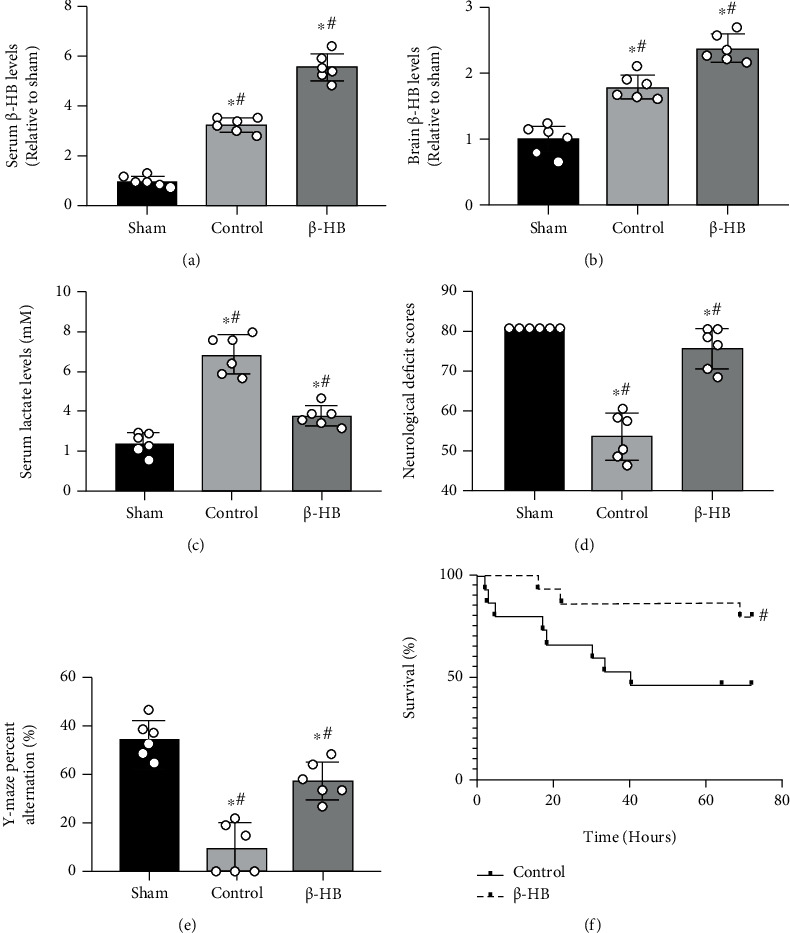
*β*-Hydroxybutyrate (*β*-HB) improved survival and neurological outcome after cardiac arrest. (a) The relative serum *β*-HB levels in rats at 6 h after return of spontaneous circulation (ROSC) (*n* = 6). (b) The relative brain *β*-HB levels in rats at 6 h after ROSC (*n* = 6). (c) *β*-HB treatment decreased serum lactate levels at 6 h after ROSC (*n* = 6). (d) Neurologic deficit scores of survived rats at 72 h after ROSC (*n* = 6). (e) Y-maze percent alternation of survived rats at 72 h after ROSC (*n* = 6). (f) *β*-HB treatment improved survival in rats within 72 h after ROSC (*n* = 15). Data are presented as mean ± SD. ^#^*p* < 0.05 versus control group. ^∗^*p* < 0.05 versus sham group.

The serum and brain *β*-HB levels at 6 hours after ROSC were measured to estimate the effect of *β*-HB on the rats. The serum and brain *β*-HB levels were both increased after ROSC (*p* < 0.05), and their levels were more significantly elevated after *β*-HB treatment compared with the control group (*p* < 0.05) (Figures 1(a) and 1(b)). Lactate is a metabolic product of anaerobic glycolysis and is an important marker of tissue injury. Our data demonstrated that the serum lactate concentrations were significantly increased at 6 hours after ROSC (*p* < 0.05), whereas its levels were significantly decreased after *β*-HB treatment (*p* < 0.05) (Figure 1(c)). To explore the effect of *β*-HB on neurological function in rats, NDS and Y-maze were performed at 72 hours after ROSC. NDSs in the *β*-HB group were significantly higher than those in the control group (*p* < 0.05) (Figure 1(d)). Moreover, the percent alternations of Y-maze in the *β*-HB group were significantly increased when compared with the control group (*p* < 0.05) (Figure 1(e)). Furthermore, the Kaplan-Meier survival analysis showed that *β*-HB treatment significantly improved the 72 hours survival time of the rats after CA (*p* < 0.05) (Figure 1(f)).

### 3.2. *β*-HB Protected against Brain Neuronal Injuries after Cardiac Arrest

**Figure 2:**
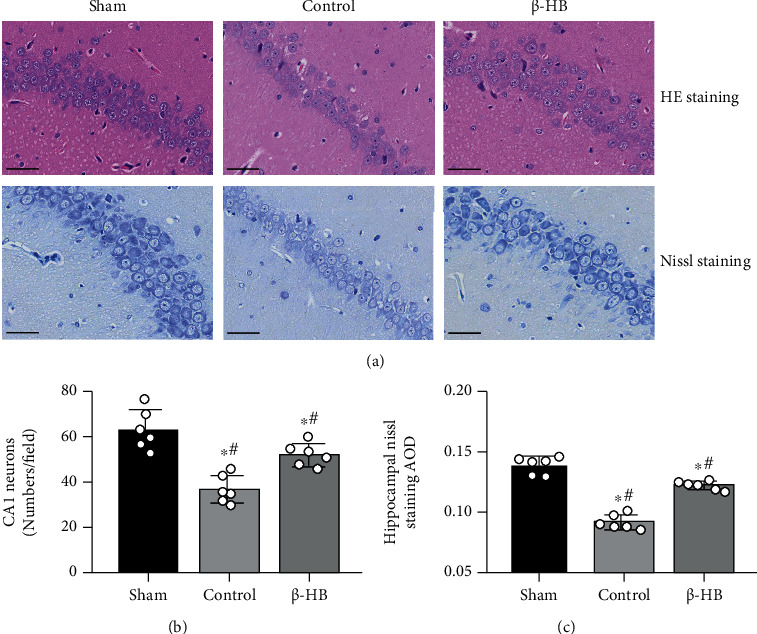
*β*-Hydroxybutyrate (*β*-HB) reduced brain histological injury after cardiac arrest. (a) Representative photograph of hematoxylin-eosin (HE) staining and Nissl staining in hippocampal CA1 neurons at 24 h after return of spontaneous circulation (×400). Neuronal cells of sham group arrange more orderly with more Nissl bodies than control group and *β*-HB group. Scale bar = 50 *μ*m. (b) Quantification of neurons in hippocampal CA1 area by HE staining (*n* = 6). (c) Average optical density (AOD) of neurons in hippocampal CA1 area by Nissl staining (*n* = 6). Data are presented as mean ± SD, ^#^*p* < 0.05 versus control group. ^∗^*p* < 0.05 versus sham group.

HE staining and Nissl staining were performed at 24 hours after ROSC to examine the effects of *β*-HB on neural injury in rat hippocampal CA-1 area. The neurons in the hippocampal CA-1 area of the control group were disorder arrangement compared with the sham group, but those of the *β*-HB group were tightly arranged (Figure 2(a)). The HE staining showed that the number of neurons significantly decreased in the control group compared with the sham group (*p* < 0.05), whereas the number in the *β*-HB group significantly increased compared with the control group (*p* < 0.05) (Figure 2(b)). Nissl staining revealed that the AOD of neurons in the hippocampal CA1 area in the control group was lower in comparison with the sham group (*p* < 0.05). However, *β*-HB treatment could significantly increase the AOD of neurons (*p* < 0.05) (Figure 2(c)).

### 3.3. *β*-HB Decreased Brain Oxidative Stress after Cardiac Arrest

**Figure 3:**
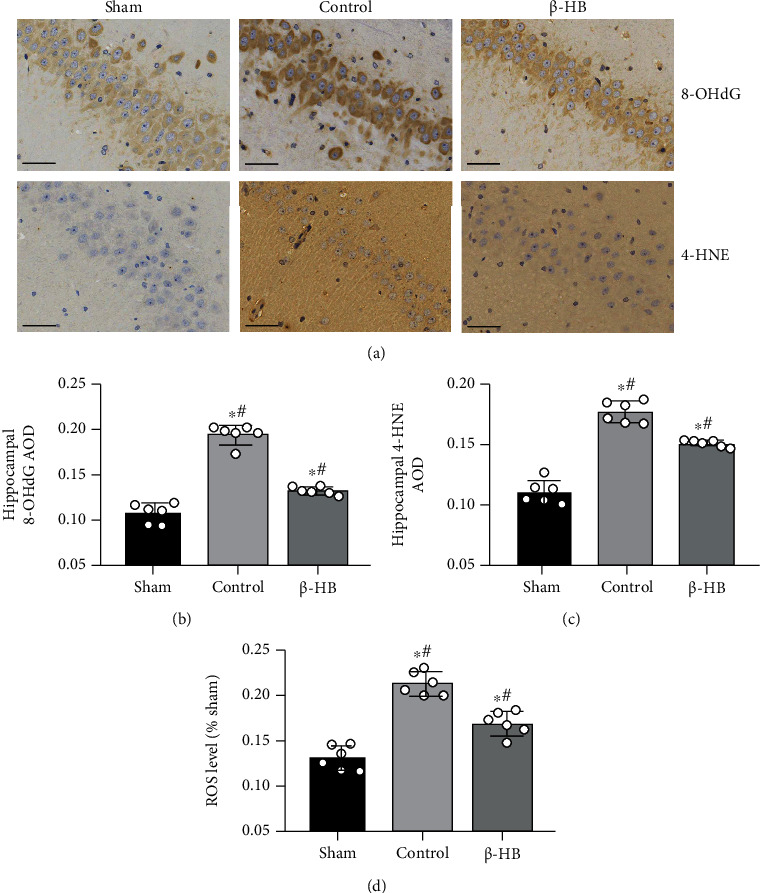
*β*-Hydroxybutyrate (*β*-HB) decreased brain oxidative stress after cardiac arrest. (a) Representative photographs of 8-hydroxy-2′-deoxyguanosine (8-OHdG) and 4-hydroxynonenal (4-HNE) immunostaining in hippocampal CA1 neurons at 24 h after return of spontaneous circulation (ROSC) (×400). Scale bar = 50 *μ*m. (b) Average optical density (AOD) of neurons in hippocampal area by 8-OHdG immunostaining (*n* = 6). (c) AOD of neurons in hippocampal area by 4-HNE immunostaining (*n* = 6). (d) The relative levels of reactive oxygen species (ROS) in brain tissues at 6 h after ROSC (*n* = 6). Data are presented as mean ± SD. ^#^*p* < 0.05 versus control group. ^∗^*p* < 0.05 versus sham group.

To examine the effect of *β*-HB on brain oxidative stress at 24 hours after ROSC, immunohistochemistry for 8-OHdG (an index of oxidative DNA damage) and 4-HNE (the end product of lipid peroxidation) was performed. The 8-OHdG positive cells and 4-HNE positive neurons were distributed throughout the hippocampus after ROSC (Figure 3(a)). Compared with the control group, AODs of 4-HNE and 8-OHdG in the hippocampal area were obviously lower in the *β*-HB group (*p* < 0.05) (Figures 3(b) and 3(c)). Furthermore, we tested the brain ROS levels of the three groups at 24 hours after ROSC. The brain ROS levels significantly increased at 6 hours after CA (*p* < 0.05), but its levels decreased significantly after treatment with *β*-HB (*p* < 0.05) (Figure 3(d)). These results suggested that *β*-HB suppressed the brain oxidative stress after CA.

### 3.4. *β*-HB-Protected Neurons from Apoptosis after Cardiac Arrest

**Figure 4:**
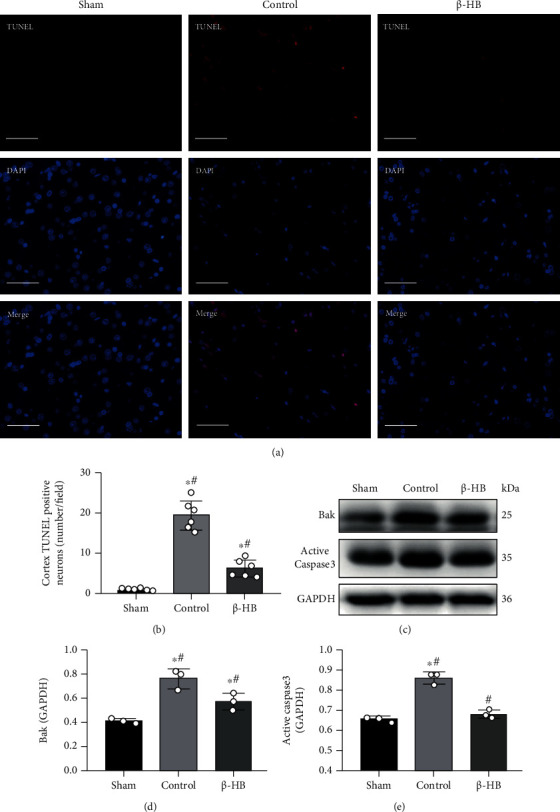
*β*-Hydroxybutyrate (*β*-HB) decreased neuronal apoptosis after cardiac arrest. (a) Representative photograph of terminal deoxynucleotide transferase-mediated dUTP-biotin nick-end labeling (TUNEL) in brain at 24 h after return of spontaneous circulation (ROSC) (×400). Scale bar = 50 *μ*m. (b) Quantification of TUNEL-positive neurons in brain after ROSC (*n* = 6). (c–e) *β*-HB treatment significantly decreased Bak and active caspase 3 protein expression levels at 24 h after ROSC (*n* = 3). Data are presented as mean ± SD. ^#^*p* < 0.05 versus control group. ^∗^*p* < 0.05 versus sham group.

The TUNEL assay showed that the TUNEL-positive neurons in the cortex area significantly increased after CA compared to the sham group, and this increase was attenuated by *β*-HB treatment (*p* < 0.05) (Figures 4(a) and 4(b)). To further assess apoptosis in the brain, Bak and active caspase 3 protein expressions were measured at 24 hours after ROSC. The Bak and active caspase 3 protein expressions were significantly increased after CA, whereas their expressions were inhibited by *β*-HB treatment (*p* < 0.05) (Figures 4(c)–4(e)).

### 3.5. *β*-HB-Protected Neurons from Pyroptosis after Cardiac Arrest

**Figure 5:**
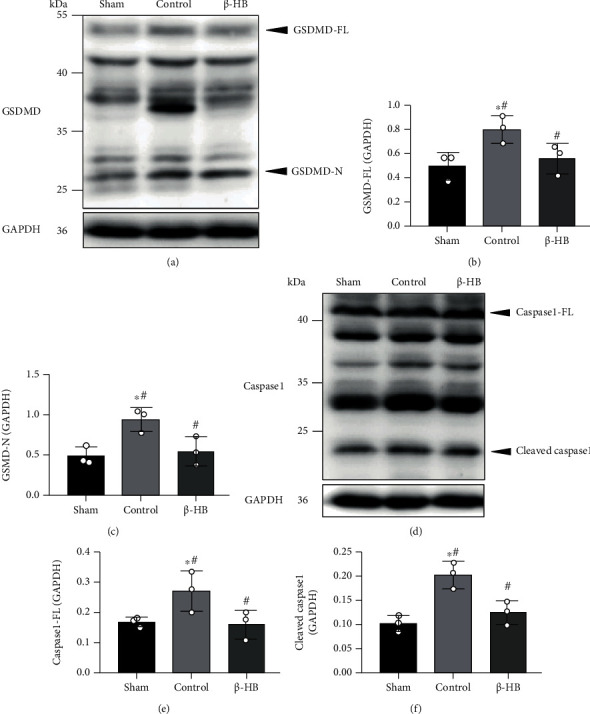
*β*-Hydroxybutyrate (*β*-HB) decreased neuronal pyroptosis after cardiac arrest. (a–c) *β*-HB treatment decreased full-length GSDMD (GSDMD-FL) and N-terminal fragment of GSDMD (GSDMD-N) protein expression levels at 24 h after return of spontaneous circulation (ROSC) (*n* = 3). (d–f) *β*-HB decreased caspase 1-FL and cleaved caspase 1 protein expression levels at 24 h after ROSC (*n* = 3). Data are presented as mean ± SD. ^#^*p* < 0.05 versus control group. ^∗^*p* < 0.05 versus sham group.

Except apoptosis, we also explored another type of cell death, pyroptosis. We detected the protein expressions related to pyroptosis at 24 hours after CA, including GSDMD and caspase 1. Pyroptosis usually refers to GSDMD-mediated pyroptosis, in which full-length GSDMD (GSDMD-FL) is cleaved into a C-terminal fragment and an N-terminal fragment (GSDMD-N), the active fragment [13]. As the results showed, the protein expressions of GSDMD-FL and GSDMD-N both increased significantly in the control group as compared with those in the sham group (*p* < 0.05). However, *β*-HB treatment decreased protein expressions of GSDMD-FL and GSDMD-N (*p* < 0.05) (Figures 5(a)–5(c)). Caspase 1 can be cleaved into p20 and p10 subunits, and these two subunits were combined to generate a tetramer, which is the active species [14]. Our study found that the expressions of the full-length p46 and the cleaved p20 fragment both significantly increased after CA. However, treatment of *β*-HB significantly decreased the expressions of full-length and cleaved caspase 1 (*p* < 0.05) (Figures 5(d)–5(f)).

### 3.6. *β*-HB Inhibited Brain Neuronal Mitochondrial Fission and Improved Mitochondrial Function after Cardiac Arrest

**Figure 6:**
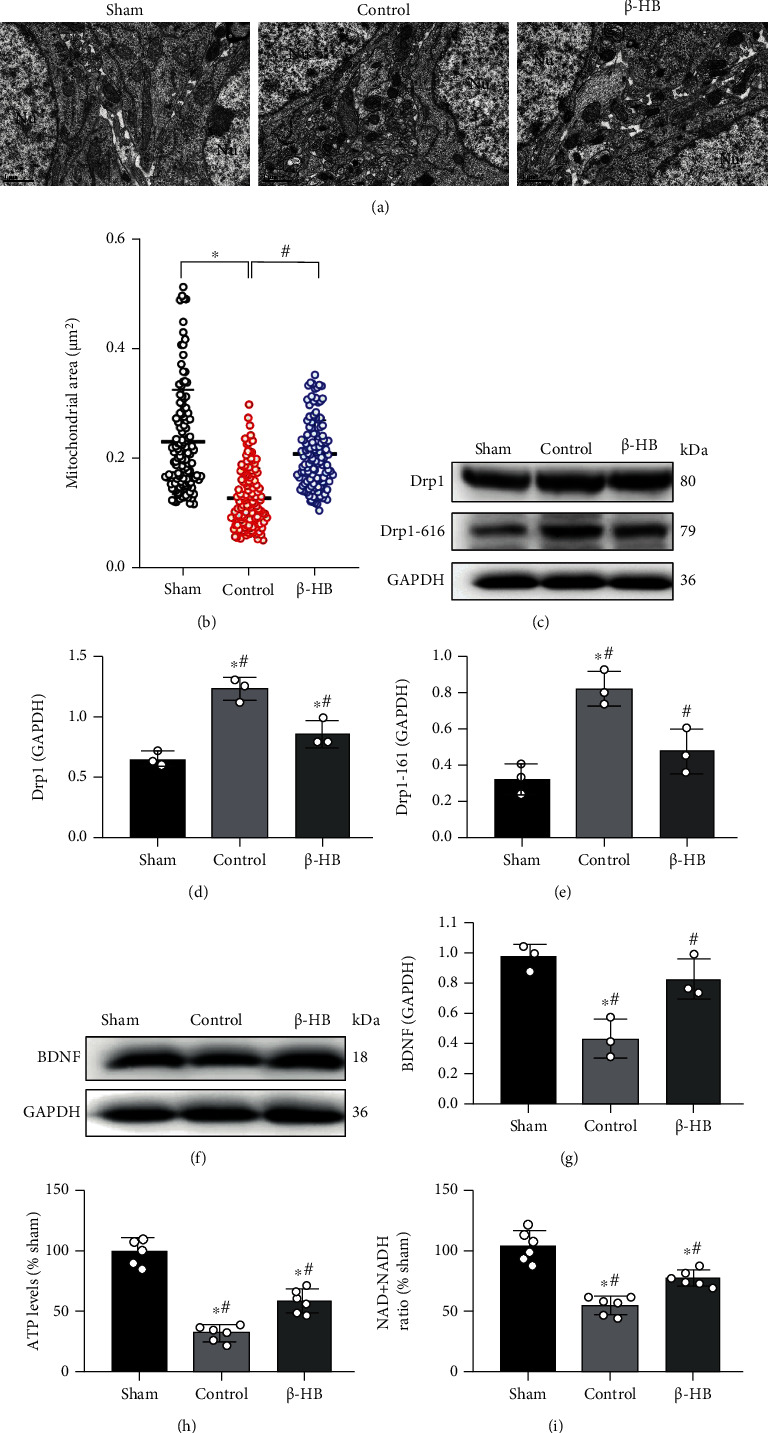
*β*-Hydroxybutyrate (*β*-HB) decreased mitochondrial fission and improved brain mitochondrial function after cardiac arrest. (a) Representative transmission electron microscopy (TEM) images of brain mitochondria at 6 h after return of spontaneous circulation (ROSC). The neuronal nucleus (Nu) is surrounded by relatively uniform and compact mitochondria. Scale bar = 1 *μ*m. (b) Quantification of brain mitochondrial area by TEM (*n* = 3) and at least 40 mitochondria were counted in each group. (c–e) *β*-HB treatment decreased Drp1 and p-Drp1 616 protein expression levels at 24 h after ROSC (*n* = 3). (f, g) *β*-HB treatment increased brain-derived neurotrophic factor (BDNF) protein expression levels at 24 h after ROSC (*n* = 3). (h) *β*-HB treatment increased brain adenosine triphosphate (ATP) levels at 24 h after ROSC (*n* = 6). (i) *β*-HB treatment increased brain NAD^+^/NADH ratio at 24 h after ROSC (*n* = 6). Data are presented as mean ± SD. ^#^*p* < 0.05 versus control group. ^∗^*p* < 0.05 versus sham group.

The structure and integrity of mitochondria are important to the physiological function of brain [15]. To investigate the effect of *β*-HB on mitochondrial ultrastructure after CA, the mitochondria in neurons at 6 hours after ROSC were observed by transmission electron microscopy. The electron microscopy analysis showed that the sizes of mitochondria were smaller in the control group than those in the sham group (*p* < 0.05) (Figures 6(a) and 6(b)). However, *β*-HB treatment significantly increased the area of mitochondria (*p* < 0.05) (Figures 6(a) and 6(b)). To explore whether the effect of *β*-HB on mitochondrial structure was associated with mitochondrial fission, we examined the protein expression that regulated mitochondrial fission and Drp1 and Drp1 phosphorylation at serine 616 (p-Drp1 616). The results of Western blot showed that the expressions of Drp1 and p-Drp1 616 significantly increased after CA, but these expressions were inhibited by *β*-HB treatment (*p* < 0.05) (Figures 6(c)–6(e)). BDNF is an essential protein to maintain quality and function of mitochondria [16], so we next examined the expression of BDNF. As expected, the expression of BDNF was significantly decreased after CA, whereas treatment of *β*-HB increased the expression of BDNF compared with the control group (*p* < 0.05) (Figures 6(f) and 6(g)). In addition, to determine whether there were beneficial effects of *β*-HB in improving mitochondrial function after CA, we detected brain ATP levels and brain NAD^+^/NADH ratio at 6 hours after ROSC. The results showed that the ATP levels and NAD^+^/NADH ratio in the *β*-HB group were significantly increased compared with the control group (*p* < 0.05) (Figures 6(h) and 6(i)). Therefore, these results suggested that *β*-HB treatment inhibited mitochondrial fission and increased mitochondrial function.

## 4. Discussion

In the present study, the neuroprotective role of *β*-HB associated with mitochondrial function and the potential mechanisms were explored. Our results indicated that *β*-HB treatment reduced the brain pathological damage, improved the neurological outcome, and significantly increased survival time at 72 hours after CA. *β*-HB also ameliorated CA-induced neurological injury by reducing cell apoptosis and pyroptosis. The possible mechanism is that *β*-HB could suppress mitochondrial fission and then alleviate mitochondrial dysfunction.

Mitochondria are the primary organelles that are especially plentiful in the brain to supply cells with energy. Imbalance between mitochondrial fission and fusion always causes mitochondrial structural changes and dysfunction [17]. The increased fission or decreased fusion results in the fragmentation of mitochondria [18]. Early studies have shown that inhibition of mitochondrial fission reduced mitochondria-related apoptosis and enhanced myocardial and neurological outcomes after CA [15]. Increasing numbers of studies improve that Drp1 is a crucial regulator in the mitochondrial fission process [19]. Posttranslational modifications are indispensable for regulating Drp1 activity in biological dynamics. Posttranslational modifications of Drp1 mainly include phosphorylation, S-nitrosylation, SUMOylation, ubiquitination, and O-GlcNAcylation [20]. As the phosphorylation of Drp1 is initiated at several sites, it participates in different mechanisms of varieties of pathological processes. In general, phosphorylation at Ser-616 activates Drp1 activity while phosphorylation at Ser-637 inhibits Drp1 activity [21].

BDNF is initially identified as a development factor generated in the central nervous system, which promotes neuron growth, cell survival, and neural delivery. Moreover, it has been proved to be the most abundant neurotropic growth factor of the hippocampus and is responsible for learning and memory [22]. In addition to its neurotrophic effects, BDNF has been proved to protect the mitochondrial structure and function [16]. Trk receptors are a family consisting of three receptor tyrosine kinases, which can be activated by neurotrophins, such as BDNF. Trk receptor activation leads to phosphorylation of several evolutionarily conserved tyrosines and results in the activation of intracellular signaling events including Ras-Raf-Erk, PI3 kinase-Akt, and atypical protein kinase C pathways [23]. Bax is a proapoptotic protein of the Bcl-2 protein family, and it is reported that the completion of its recruitment to the mitochondrial outer membrane (MOM) regulates mitochondrial fission [24]. However, the activation of PI3 kinase-Akt pathway prevents Bax translocating to MOM and, thus, inhibits mitochondrial fission [25]. Therefore, BNDF inhibits mitochondrial fission through activating Trk receptors and PI3 kinase-Akt pathway.

To investigate the neuroprotective effect of *β*-HB treatment after CA, we evaluated the size of mitochondria using transmission electron microscopy. The results showed that treatment of *β*-HB significantly increased the mitochondrial area, suggesting a reduction of mitochondrial fission. Furthermore, we found that *β*-HB treatment downregulated the expression of Drp1 and p-Drp1 616, whereas upregulated the expression of BDNF. These results indicated that *β*-HB treatment inhibited mitochondrial fission.

As what we have discussed before, the structural completeness of mitochondria is important to the mitochondrial function. Since *β*-HB could reduce mitochondrial fission, we assumed that it could improve mitochondrial function and increase neurological energy metabolism. The energy supply of the normal brain relies on complete oxidation of glucose. Global cerebral ischemia results in decrease of oxidative phosphorylation and increase of glycolysis. However, this incomplete oxidation of glucose not only cannot produce enough ATPs but also leads to the accumulation of lactate. When glucose oxidation fails to provide sufficient acetyl-CoA for Krebs cycle, ketogenesis is promoted to produce KBs [26]. Then, *β*-HB is transported to brain mitochondria and is metabolized back into acetyl-CoA. Thus, as a replacement of fuel, *β*-HB improves energy metabolism and reduces the brain injury [27]. In order to evaluate the effect of *β*-HB on brain energy metabolism, we detected the levels of ATP and NAD^+^/NADH ratio of brain tissue at 6 hours after ROSC. We found that both the levels of ATP and NAD^+^/NADH ratio were significantly decreased after CA, whereas *β*-HB treatment increased these levels. Thus, our results suggested that *β*-HB treatment improved energy supply after CA through inhibiting mitochondrial fission.

Mitochondria play a crucial role in the production of ROS except for the generation of ATP. Previous studies have proved that mitochondrial fission is associated with increased ROS production [28]. Overgeneration of ROS has been linked to acute brain injury [29]. Oxidative stress is defined as the imbalance between the creation of ROS and the ability to clear reactive intermediates. At high levels, ROS changes cellular metabolism and the internal environment, leading to apoptosis. According to recent evidence, excessive ROS generation can lead to the functional and structural damage of neurons [30]. Intracellular ROS induces the oxidation of large numbers of molecules, for example, DNA, lipids, and proteins [31]. 8-OHdG and 4-HNE are oxidative stress makers that predict the degree of oxidative damage to DNA and lipids [32]. Here, we found that 8-OHdG and 4-HNE positive neurons were significantly increased after CA, indicating high oxidative stress in brain. As expected, *β*-HB treatment suppressed the oxidative stress in brain after CA. Consistent with a previous study, our study demonstrated that the neuroprotection of *β*-HB was through suppressing mitochondrial fission and oxidative stress.

Apoptosis is defined as procedural cell death, which is a mechanism to clean unnecessary neurons and represents a physiological and protective reaction to neurological damage. However, increasing studies indicated that excessive activation of apoptosis may be harmful, peculiarly in pathological conditions [33]. Under I/R injury conditions, mitochondrial fission plays a main role in cellular apoptosis [34]. Drp1 is an important mitochondrial fission protein, which is upregulated and translocated from the cytosol to mitochondrial in I/R conditions, followed by increased mitochondrial fission and cell apoptosis [35]. Moreover, mitochondria are crucial to apoptosis through releasing proapoptotic factors from the intermembrane space into the cytoplasm. The Bal-2 protein family is stored in the mitochondrial intermembrane and plays an important role in apoptosis. Bak is one of the proapoptotic proteins from Bal-2 family [36]. Caspase 3 is the downstream response factor of Bak in apoptotic-associated pathway. Our results showed that the expressions of Drp1, Bak, and caspase 3 significantly increased after CA. However, treatment of *β*-HB ameliorated the neurological apoptosis after CA.

As we have discussed above, mitochondria are the major source of ROS, and excessive mitochondrial fission results in aberrant ROS accumulation. Previous research has reported the mitochondrial ROS-Txnip-Nlrp3 pathway in diabetic nephropathy. As a response to different kinds of exogenous and endogenous stimuli, the Nlrp3 inflammasome induces caspase 1-dependent pyroptosis [37]. Pyroptosis is a unique kind of programmed cell death which is different from apoptosis and necrosis. Caspase 1 is a main molecule that causes the induction of pyroptosis by cleaving GSDMD into the active amino-terminal domain (GSDMD-N) and the inhibitory carboxy-terminal domain (GSDMD-C) [38]. As a result, the GSDMD-N domain oligomerizes and inserts into the plasma membrane, leading to a rapid loss of plasma membrane and cell lysis, eventually causing neuron death [39]. Accumulating studies have showed that *β*-HB has antioxidative and anti-inflammatory effects, and exogenous *β*-HB has medicinal effects in pathological conditions, such as cerebral hypoxia, anoxia, and ischemia [40]. A possible mechanism where KB has a beneficial role against brain injury is its influence on pyroptosis. Our results showed that not only the expression of full length of caspase 1 and GSDMD but also the expression of their cleaved active domain increased at 24 hours after CA, whereas treatment of *β*-HB reduced these expressions and protected neurological cell death from pyroptosis.

Taken together, our study suggested that *β*-HB treatment improved neurological outcomes after CA through maintaining mitochondrial function by inhibiting mitochondrial fission. We suppose that mitochondrial-targeted therapy is a potential intervention in brain injury after CA, and *β*-HB is a hopeful candidate for treatment. However, determination of the optimum dose and optimal treatment time is important to achieve the best therapeutic benefits of *β*-HB. At proper concentrations, *β*-HB shows cytoprotective effects and can activate obvious endogenous cell defense pathways, while at high concentrations, it may induce cytotoxicity. Furthermore, the treatment of *β*-HB in our study were given after ROSC, the effects of *β*-HB treatment before CA are still unknown. Therefore, further studies are necessary to optimize the therapeutic dose and treatment time of *β*-HB for neurological injury after CA to carry out more available therapeutic effects.

## 5. Conclusions

The present study demonstrated that administration of *β*-HB improves neurological outcomes and enhances survival time after CA. Moreover, this study suggests that the potential mechanism of the neuroprotective effects of *β*-HB is the inhibition of mitochondrial fission. The *β*-HB treatment plays a role in possible therapeutic application after CA and serves as a hopeful treatment for neurological function in post-CA patients.

## Data Availability

The data used to support the findings of this study are available from the corresponding authors upon request.
